# Untargeted and Targeted Metabolomic Profiling of Preterm Newborns with EarlyOnset Sepsis: A Case-Control Study

**DOI:** 10.3390/metabo11020115

**Published:** 2021-02-18

**Authors:** Veronica Mardegan, Giuseppe Giordano, Matteo Stocchero, Paola Pirillo, Gabriele Poloniato, Enrica Donadel, Sabrina Salvadori, Carlo Giaquinto, Elena Priante, Eugenio Baraldi

**Affiliations:** 1 Women’s and Children’s Health Department, Padua University, 35128 Padua (PD), Italy; giuseppe.giordano@unipd.it (G.G.); matteo.stocchero@unipd.it (M.S.); paola.pirillo@gmail.com (P.P.); poloniato.gabriele@gmail.com (G.P.); enrica.donadel@gmail.com (E.D.); sabrina.salvadori@aopd.veneto.it (S.S.); carlo.giaquinto@unipd.it (C.G.); elena.priante@aopd.veneto.it (E.P.); eugenio.baraldi@unipd.it (E.B.); 2 Fondazione Istituto di Ricerca Pediatrica Città della Speranza, 35100 Padua (PD), Italy; 3 Laboratory of Mass Spectrometry and Metabolomics, Women’s and Children’s Health Department, Padua University, 35100 Padua (PD), Italy

**Keywords:** sepsis, earlyonset sepsis, infant/newborn, metabolomics, biomarker

## Abstract

Sepsis is a major concern in neonatology, but there are no reliable biomarkers for its early diagnosis. The aim of the study was to compare the metabolic profiles of plasma and urine samples collected at birth from preterm neonates with and without earlyonset sepsis (EOS) to identify metabolic perturbations that might orient the search for new early biomarkers. All preterm newborns admitted to the neonatal intensive care unit were eligible for this proof-of-concept, prospective case-control study. Infants were enrolled as “cases” if they developed EOS, and as “controls”if they did not. Plasma samples collected at birth and urine samples collected within 24 h of birth underwent untargeted and targeted metabolomic analysis using mass spectrometry coupled with ultra-performance liquid chromatography. Univariate and multivariate statistical analyses were applied. Of 123 eligible newborns, 15 developed EOS. These 15 newborns matched controls for gestational age and weight. Metabolomic analysis revealed evident clustering of the cases versus controls, with the glutathione and tryptophan metabolic pathways markedly disrupted in the former. In conclusion, neonates with EOS had a metabolic profile at birth that clearly distinguished them from those without sepsis, and metabolites of glutathione and tryptophan pathways are promising as new biomarkers of neonatal sepsis.

## 1. Introduction

Neonatal sepsis is an infection-induced, systemic inflammatory response syndrome common in premature and term neonates [1,2,3]. It is one of the leading causes of neonatal death and morbidity and is believed to have a key role in most inflammatory disorders that cause or enhance the main morbidities affecting the preterm (bronchopulmonary dysplasia, white matter injury, necrotizing enterocolitis, and retinopathy of prematurity) [3,4,5,6,7,8]. Sepsis in the newborn is typically classified as either earlyonset sepsis (EOS), when the infection occurs within three days after birth, or late-onset sepsis (LOS) if it develops afterward [9,10]. Early detection of neonatal sepsis and prompt administration of broad-spectrum antibiotic therapy can prevent its clinical course towards septic shock and death, but it is not easy to diagnose neonatal sepsis early on. Blood culture is still considered the gold standard, even though it takes time to obtain the results, and false-negative findings are not uncommon because neonatal bacteremia is often intermittent, and intrapartum antibiotic treatment may limit the culture’s diagnostic value [11,12,13,14,15,16]. Neonatal sepsis is therefore mainly suspected on the grounds of non-specific clinical signs and symptoms; moreover, none of the most widelyused biomarkers are entirely reliable indicators of sepsis in newborns [9,11,17]. Hence, identifying new biomarkers for EOS is of crucial importance. Furthermore, while supportive therapies promote the survival of septic neonates, no mechanistic therapies exist to alter the underlying pathophysiology, and this is partly due to the ignorance of the complex biological pathways underlying the pathophysiology of sepsis.

Metabolomics is a high-dimensional technology that studies the complete set of low molecular weight metabolites present at a certain time in a complex biological system using body fluids such as urine, blood, or stool. It is considered the “-omic” platform most closely related to host phenotype [18]. Because sepsis is the result of a complex interaction between host and pathogen, and the impact of infectious perturbations on the metabolome includes highlydynamic, short-lived changes, metabolomics is a more suitable approach for studying sepsis than more “static” genomics, transcriptomics, or proteomics. Metabolomics can therefore help in improving our knowledge of the complex and dynamic mechanisms underlying sepsis and has already been used in adult patients and children with severe infections [19,20,21,22,23,24,25,26,27].

Different approaches are used in metabolomics. The untargeted approach aims to analyze samples without any a priori hypothesis on the metabolic pathways associated with a given pathological condition. It does not necessarily involve identifying each metabolite but tries to detect the metabolic fingerprint that discriminates between groups of subjects. The search for these metabolic patterns is not driven by a priori hypothesis, so it is open to new findings and unexpected metabolites may turn out to be important in characterizing specific groups of subjects so new pathophysiological hypotheses may be formulated and new diagnostic or prognostic biomarkers may emerge [18]. On the other hand, the targeted metabolomic approach aims to quantify a set of metabolites belonging to specific metabolic pathways or a well-defined class of chemical compounds [18].

The ultimate goal of our research program is the discovery of early biomarkers of neonatal sepsis, and in the present prospective case-control study, a two-step metabolomic approach based on mass spectrometry was applied to neonates affected by EOS in an effort to investigate the perturbations at the metabolome level that might lead to the discovery of novel early biomarkers of this condition. Firstly, an untargeted metabolomic approach was used to compare the metabolic profiles of urine samples collected within 24 h of birth from preterm neonates with and without EOS. Then, the results of this untargeted metabolomic analysis were used to guide a targeted metabolomic examination of plasma samples collected at birth from the same neonates to investigate and validate the metabolic derangements induced by sepsis further.

## 2. Results

Among 123 eligible neonates, 15 met the criteria for earlyonset sepsis (EOS group), and 15 without sepsis matched for gestational age and weight were enrolled as controls. The EOS group had a gestational age of 207 (17) days, and a birth weight of 1269 (358) g, while the control group had a gestational age of 213 (16) days, and a birth weight of 1300 (354) g. The two groups did not show significant differences in gestational age (*p*-value = 0.34) and weight (*p*-value = 0.82) on the basis of the t-test. Moreover, none of the infants died, and they were all discharged home. In the EOS group, one blood culture tested positive (*Streptococcus agalactiae*); the other neonates were classified as septic infants on the basis of clinical signs (cardiovascular or respiratory instability, neurologic signs) and laboratory findings obtained within 72 h from birth (leukopenia, leukocytosis, increased C-reactive protein, increased serum lactate), in accordance with the criteria established in 2010 at an expert meeting of the European Medicines Agency on neonatal and pediatric sepsis [28]. The average time point for sepsis diagnosis was 25 h of life.

Urine samples were collected within 24 h after birth for 9 cases and 10 controls. Table 1 shows the recruited neonates’ demographic and perinatal characteristics, and laboratory findings at birth—no significant differences emerged between the EOS and control groups.

While laboratory findings at birth showed no differences between the two groups, C-reactive protein value at 24–72h from birth was significantly higher in the EOS group (CRP 26 [16] mg/L) than in the control group (CRP 2.3 [1.6] mg/L) on the basis of the Mann–Whitney test (*p*-value < 0.001). All neonates recruited as controls did not manifest any infection within seven days of birth.

In a first step, untargeted metabolic profiling of urine samples was applied to discover which are metabolic pathways perturbed by sepsis. These pathways were then analyzed more in depth in a second step using targeted methods on plasma.

Two data sets were obtained in the untargeted metabolic step. Specifically, a data set including 2394 RT_mass variables was generated by the negative ionization mode (indicated as NEG data set in the following), and a data set with 3224 RT_mass variables was obtained using the positive ionization mode (POS data set). No outliers were detected by principal component analysis (PCA) on the basis of the T2 and Q tests (see Section S1.4 in Supplementary Materials for more details).

Univariate data analysis based on t-test or Mann–Whitney test with false discovery rate highlighted 44 variables in the NEG data set, and 332 in the POS data set as relevant (δ = 0.10), for a total of 376 relevant features.

**Figure 1:**
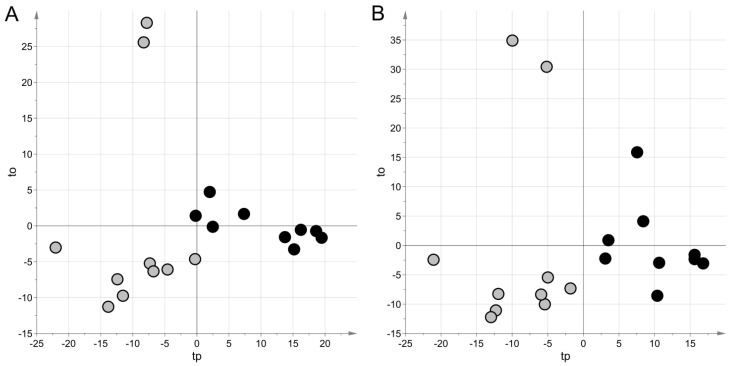
Untargeted metabolic profiling: score scatter plots obtained with projection to latent structures discriminant analysis(PLS–DA) modeling for urine samples. Samples from cases of sepsis are indicated with black circles, those from controls with light grey circles; panel (**A**) NEG data set; panel (**B**) POS data set. The PLS–DA models have been post-transformed to obtain the predictive latent variable tp and the non-predictive latent variable to [29].

For the NEG data set, projection to latent structures discriminant analysis (PLS–DA) generated a model with two latent variables, a Matthew correlation coefficient in calculation (MCC) of 0.90 (*p* = 0.038), and an MCC calculated by five-fold cross-validation (MCC_5-fold_) of 0.49 (*p* = 0.023). For the POS data set, a model with two latent variables, MCC = 0.90 (*p*-value = 0.042) and MCC_5-fold_ = 0.37 (*p*-value = 0.034), were obtained. Figure 1 shows the score scatter plots obtained with the models. It is worth noting that samples from the same group clustered in the same region of the plot, according to the high values of MCC.

Stability selection has been implemented to highlight the relevant features from a multivariate point of view. All the 210 features discovered as relevant resulted to belong to the set of 376 relevant features discovered by univariate data analysis. Thus, the relevant variables were submitted to the annotation process. Overall, 60 variables were annotated [30]—14 at level 1 (“identified metabolites”); 44 at level 2 ("putatively annotated compounds"); and 2 at level 3 ("putatively characterized compound classes"). The annotated variables are reported in Table 2.

**Figure 2:**
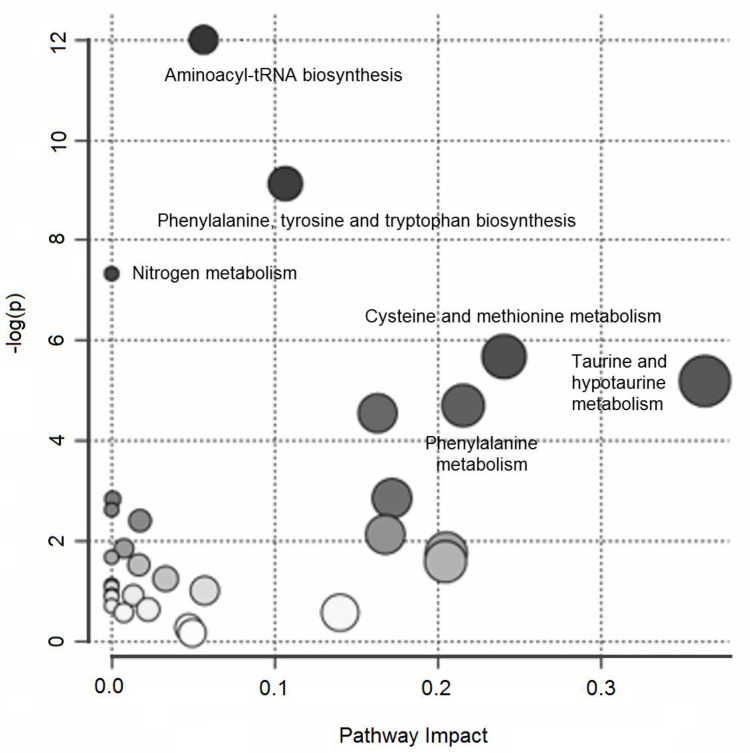
Untargeted metabolic profiling of urine samples: over-representation pathway analysis. The impact of each perturbed pathway is shown against its negative log *p*-value (-log(*p*)). The names of the pathways with a *q-*value less than 0.15 are shown in the figure.

Over-representation pathway analysis conducted on the annotated variables revealed six perturbed pathways (δ = 0.15) related to aminoacyl-tRNA biosynthesis; phenylalanine, tyrosine, and tryptophan biosynthesis; nitrogen metabolism; cysteine and methionine metabolism; taurine and hypotaurine metabolism; and phenylalanine metabolism. Figure 2 summarizes the results of the over-representation pathway analysis.

In the second step of our metabolomic investigation, 64 metabolites closely related to the perturbed pathways were quantified in the blood samples using targeted metabolomics to confirm the findings of the previous untargeted step (see Section S2 and Tables S1 and S2 in Supplementary Materials for more details). Specifically, 40 metabolites belonging to the families of amino acids, nine neurotransmitters associated with tyrosine and tryptophan metabolism, and six polyamines and nine metabolites associated with the kynurenine pathway were quantified. Univariate data analysis identified 26 metabolites as relevant (δ = 0.10).

**Figure 3:**
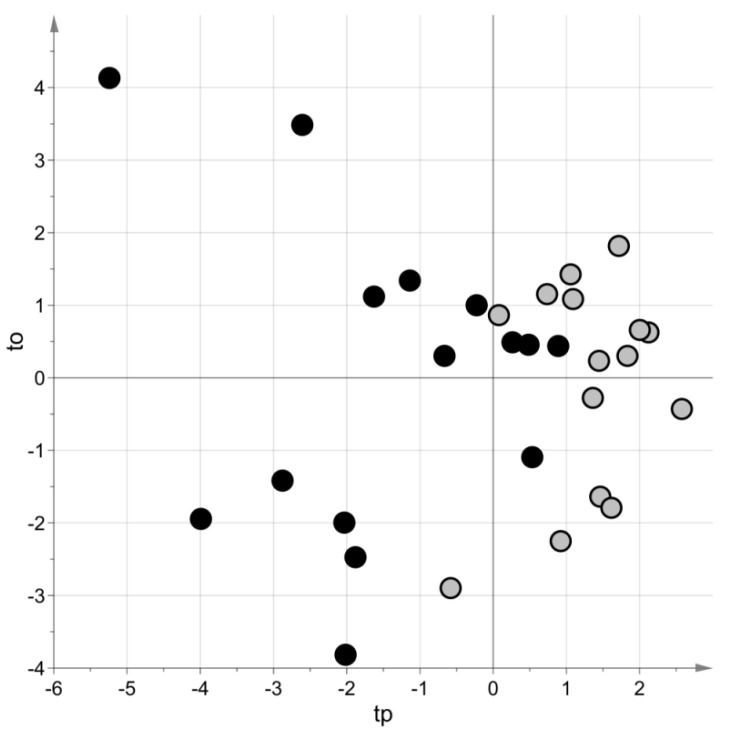
Targeted metabolomic investigation: score scatter plot of the PLS–DA model built using the blood samples. Samples from cases of sepsis are indicated with black circles, those from controls with light grey circles. The PLS–DA model has been post-transformed to obtain the predictive latent variable tp and the non-predictive latent variable to [29].

PLS–DA generated a model with two latent variables, MCC = 0.69 (*p*-value = 0.08) and MCC_5-fold_ = 0.62 (*p*-value = 0.009). Figure 3 shows the score scatter plot obtained with the model. Blood samples of the same group belong to the same region of the plot.

Stability selection led to four metabolites being selected as relevant for the purposes of discriminating between the groups of cases and controls. These four metabolites were highlighted also by univariate data analysis. Table 3 reports the relevant metabolites arising from the targeted investigation.

**Figure 4:**
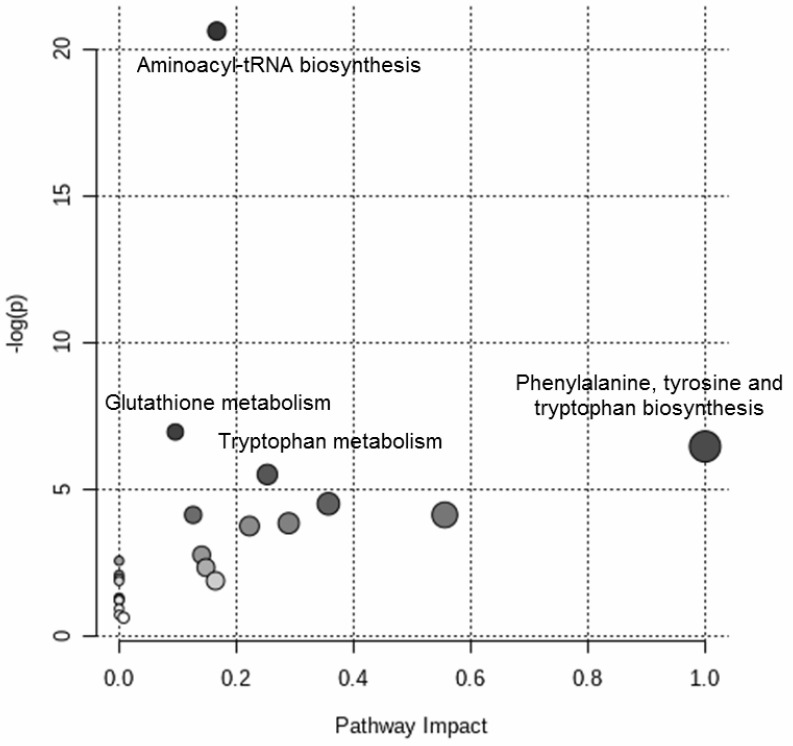
Targeted metabolomic investigation of the blood samples: over-representation pathway analysis. The impact of each perturbed pathway is shown against its negative log *p*-value (-log(*p*)). The names of the pathways with *q*-value of less than 0.10 are shown in the figure.

Figure 4 shows the results of the over-representation pathway analysis. The dysregulated pathways (δ = 0.10) were associated with aminoacyl-tRNA biosynthesis, glutathione metabolism, phenylalanine, tyrosine and tryptophan biosynthesis, and tryptophan metabolism.

## 3. Discussion

In this study, the urine and plasma metabolome of neonates with and without EOS were examined to seek perturbations that might help identify novel early biomarkers of EOS. UPLC–MS analysis of urine samples collected within 24 h after birth revealed a clear clustering of cases of EOS compared with healthy neonates. Then, a metabolic signature exists to distinguish neonates that develop sepsis and healthy subjects. Annotating the variables derived from this untargeted analysis, putative markers discriminating between the two groups (EOS cases versus controls) were discovered (Table 2). Pathway analysis shed light on the metabolic derangements most involved in EOS (Figure 2). The metabolic pathways emerging as most significant were then further investigated using a targeted analysis on plasma samples collected at birth from the same neonates, confirming the marked disruption of the tryptophan and glutathione metabolic pathways in the neonates with EOS.

Many tryptophan catabolites were enhanced in the septic neonates, and the kynurenine pathway of tryptophan metabolism was particularly stimulated. Kynurenine catabolites have both pro- and anti-oxidative properties, and they are involved in regulating glutamatergic neurotransmission and energy substrate synthesis, so tryptophan catabolism via the kynurenine pathway may have a key role in neonatal response to sepsis-induced stress [31]. Intriguingly, gut flora metabolism of tryptophan seems to be involved in metabolic anomalies induced by sepsis as well. Our results suggest that tryptophan is preferably catabolized by the gut flora of infected neonates through indole acetic acid rather than indole acrylic acid. Because the latter seems to promote the barrier function of the intestinal epithelium and mitigates inflammatory response [32,33], a decrease in this acid could exacerbate the excessive systemic inflammatory response syndrome induced by sepsis. It has been well documented that the newborns’ gut microbiome and metabolome are involved in predisposing them to sepsis and facilitating its evolution, and it can be presumed that tryptophan metabolism may be a crucial aspect of this phenomenon [34,35,36]. This is speculation and will be investigated in detail in future studies because the analysis of gut flora metabolism is beyond the aim of this study.

Glutathione metabolism also emerged as severely altered in neonates with EOS, in both untargeted and targeted analyses. This is an expected finding because glutathione is a powerful antioxidant with a key role in preventing and combating reactive oxygen species [37], which are strongly generated in septic states, and the induction of its metabolism may presumably be the result of a septic process.

Metabolomics has been used widely as a diagnostic and prognostic tool for sepsis in adults [19,20,21,22,23,24,25,26,27]. Studies on the role of metabolomics in the diagnostic work-up of pediatric and neonatal sepsis are limited, however. As explained by Mickiewicset al., we can distinguish between children with septic shock, those with non-infectious systemic inflammatory response syndrome, and healthy children from differences in their serum metabolic profiles. In addition to its diagnostic applications, the metabolomic approach has proved very accurate in establishing the prognosis for septic children [38,39]. In a case report with two control groups, Ambroggio et al. found higher urine concentrations of metabolites previously associated with sepsis in a patient with fatal methicillin-resistant *Staphylococcus aureus* pneumonia than in patients with influenza pneumonia or healthy controls. These changes in urinary metabolism preceded the clinical phenotype of severe sepsis, suggesting that establishing the extent of metabolic disruption can facilitate the early identification of a sepsis phenotype ahead of its clinical diagnosis [40].

To date, only two studies have analyzed metabolic perturbations in neonatal sepsis. In the study by Fanos et al., a combined approach based on nuclear magnetic resonance and gas-chromatography/mass-spectrometry revealed a specific urinary metabolic profile in nine neonates with sepsis (both EOS and LOS) compared with 16 healthy newborns. Despite the difference in gestational age between the groups (29.1 weeks for the cases, 34.6 weeks for the controls), this was the first study to find different metabolic profiles in neonates with and without sepsis [41]. Sarafidis et al. reported that the metabolic profiles of neonates with proven or likely LOS differed considerably from those of their healthy peers. Overall, neonates with confirmed or possible LOS exhibited comparable metabolic profiles, indicating similar metabolic alternations upon the onset of clinical manifestations [42].

As far as we know, this is the first metabolomic study in which the results of untargeted analyses on urine samples were validated using a targeted approach on plasma samples from the same neonates, which reinforces our findings. We initially analyzed urine samples because untargeted analysis requires a moderately large volume of biological fluid, and sufficient urine samples can be easily collected from preterm infants, unlike blood samples. We then confirmed the results of the first investigation by targeted analysis, which requires only a few microliters of fluid, on plasma samples collected at birth. Applying metabolomics to seeking new early biomarkers of sepsis in both urine and plasma samples offers another advantage in noninvasive urine sampling that would be particularly appropriate for neonates with only maternal risk factors or mild non-specific clinical signs of infection, who would not normally undergo blood testing. On the other hand, plasma sampling is much quicker, making it particularly useful in the sickest or most premature neonates, who would benefit most from a prompt and dependable diagnosis.

The present report also describes the first metabolomic study to focus exclusively on neonates with EOS, a condition that is a major concern for neonates born at term or preterm. Symptoms of EOS are frequently severe and take a fulminant course unless broad-spectrum antibiotic therapy is started promptly, but accurate and early biomarkers of EOS are still lacking. By revealing a clear clustering of the metabolomes of neonates with versus without EOS, our findings highlight the potential of metabolomics for discovering new early biomarkers of disease. Because the metabolism of tryptophan and glutathione was found severely disrupted in septic neonates, a few metabolites of these pathways could probably be used as biomarkers of neonatal sepsis—a hypothesis that will be investigated in future studies. Our targeted analysis revealed metabolic derangements in plasma samples obtained at birth, meaning that they occur very early on in a state of sepsis. The biomarkers emerging from this approach would therefore be very useful for the early diagnosis of EOS, which is crucial in clinical practice.

A further strength of our study lies in the low gestational age of the infants considered, which is significantly lower than in the study by Sarafidis et al. [42]. Clinical presentation is a key factor in the diagnosis of sepsis in the newborn, but the signs and symptoms are non-specific. This is particularly worrying in the case of those born very preterm, whose comorbidities may mimic sepsis. It is therefore primarily for the most premature neonates that we need to find new biochemical markers to help us diagnose sepsis. Tools for the early diagnosis of sepsis are also much needed for preterm neonates without any infections, who might be exposed to a greater risk of complications related to premature birth (e.g., LOS, necrotizing enterocolitis, reduced food tolerance) if given unnecessary antibiotic treatment.

A limitation of this study concerns the small number of infants recruited, but it reflects the relatively low incidenceof EOS. The number of infants involved in the present study is comparable with those of previouslypublished research using a metabolomic approach to the investigation of neonatal sepsis [41,42].

A second potential weakness of this report is that only one newborn in the EOS group had a positive blood culture. Although blood culture is still considered the gold standard for neonatal sepsis diagnosis, the limits of this diagnostic tool are well described, especially for the diagnosis of EOS—false-negative findings are not uncommon because neonatal bacteremia is often intermittent, and intrapartum antibiotic treatment may limit the culture’s diagnostic value [14,15,16]. To properly allocate the culture-negative neonates in the EOS group, we used the clinical and laboratory criteria identified by an expert meeting of the European Medicines Agency on neonatal and pediatric sepsis.

Finally, our results need to be confirmed, and a validation population is currently being recruited for this purpose.

Future researches will investigate, after validation of these preliminary results, the potential role of some metabolites of the tryptophan and glutathione pathways as early biomarkers of neonatal sepsis. In addition to its diagnostic accuracy in discriminating septic infants, in fact, an ideal biomarker should have many other characteristics—it should be from readily available sources, it should not be affected by comorbid conditions, and biomarker levels should vary rapidly in response to treatment, aiding in risk stratification and evaluation of prognosis. Moreover, it should be easily and rapidly measured and its analysis should not be too much expensive. If an ideal biomarker will emerge from these future researches, cost-effective methods for its measurement will be probably elaborated to fill this diagnostic gap.

## 4. Materials and Methods

### 4.1. Study Population

This prospective, case-control study was conducted at a single, tertiary-level neonatal intensive care unit (NICU) on participants recruited from December 2015 to November 2017. Preterm neonates (<37 weeks of gestation) admitted to the NICU at the Women’s and Children’s Health Department of Padua Hospital (Italy) were eligible for the study. The EOS group included any neonates classified as septic infants on the basis of clinical signs (cardiovascular or respiratory instability, neurologic signs) and laboratory findings obtained within 72 h from birth (leukopenia, leukocytosis, increased C-reactive protein, increased serum lactate), in accordance with the criteria established in 2010 at an expert meeting of the European Medicines Agency on neonatal and pediatric sepsis [28]. Specifically, the criterion was the presence of at least two clinical symptoms and at least two laboratory signs of infection. These cases of the EOS group were compared with neonates who did not manifest any infection within seven days of birth, as defined by the presence of clinical and laboratory signs, in accordance with the definition of neonatal sepsis of the expert meeting of the European Medicines Agency on neonatal and pediatric sepsis [28] (controls). To avoid any influence of gestational age and weight, each neonate diagnosed with EOS was matched with the next eligible newborn of similar gestational age and weight. Infants with major congenital or chromosomal abnormalities, or with known or suspected congenital metabolic disease, asphyxiated newborns, and those given transfusions (erythrocytes, plasma, or platelets) before any collection of samples were excluded from the study. No treatment was administered before plasma sampling. At the time of urine sampling, the same pharmacological treatment, including antibiotic therapy, and the same nutrition (both parenteral and enteral) were being administered to all neonates enrolled in the study.

### 4.2. Sample Collection

Plasma sampling: a total of 1 mL of blood collected at birth when blood tests were run on admission to the NICU (before any therapy, intravenous infusion, or milk were administered). Blood was centrifuged and the resulting plasma samples were stored at −80 °C until analysis.

Urine sampling: at least 2 mL of urine was collected noninvasively within 24 h of birth, by placing a cotton ball inside the newborn’s nappy and checking for the presence of urine every 30 min. The cotton ball was changed if the neonate did not urinate within 3 h of its placement or if it was contaminated with fecal material. After the neonate urinated, the cotton ball was placed in the barrel of a syringe and squeezed with the plunger to collect the absorbed urine in a container prewashed with MeOH, for metabolomic analysis. The same brands of nappies and cotton balls were used throughout the study. Samples were stored at −80 °C until analysis.

### 4.3. Metabolomic Analysis

The analysis was performed at the Mass Spectrometry and Metabolomics Laboratory of the University of Padua’s Women’s and Children’s Health Department.

Urine samples were slowly thawed to ambient temperature. Each sample was stirred and centrifuged at 3600 g, then 50 µL of the supernatant from each sample were pipetted in a total recovery glass vial, adding 100 µL of 0.1% formic acid solution (dilution 1:3).

Untargeted metabolic profiling was performed in positive and negative ionization mode on an Acquity ultra performance liquid chromatography (UPLC) system (Waters, Waters MS Technologies, Ltd., Manchester, UK) coupled to a quadrupole time-of-flight (QToF) Synapt G2 HDMS mass spectrometer (Waters MS Technologies, Ltd., Manchester, UK). Chromatography was performed using an Acquity HSS T3 (1.7 μm, 2.1 × 100 mm) column (Waters Corporation, Milford, CT, USA) kept at 50 °C. The flow rate of the mobile phase was set at 0.5 mL/min, and each sample run lasted 12 min, with 5 µl of the sample injected for each run. For mass accuracy, a LockSpray interface was used with a 20 μg/L leucine enkephalin. Data were collected in continuum mode, in a scanning range of 20–1200 m/z, with lock mass scans collected every 10 s and averaged over three scans for mass correction. Quality control samples (QC) and standards solution samples (Mix) were used to assess reproducibility and accuracy during the analysis, and examine the metabolite content of the samples. The QCs were prepared from an aliquot of each sample, diluted with 0.1% formic acid solution with three different dilution factors (1:3, 1:5, and 1:7). The mix consisted of nine compounds of known exact mass and retention time. The QCs and mixes were injected at regular intervals during the sequence, together with blank samples to identify specific ions from the mobile phase, and any contaminants. Samples were injected randomly to prevent any spurious classification deriving from the position of the sample in the sequence. Data were pre-processed using Progenesis software (Waters Corporation, Milford, CT, USA). The parameters used for data extraction were optimized through the preliminary analysis processing of the QC samples. The ion intensities for each peak detected were normalized, based on the calibration models obtained for the QCs with different dilution factors (1:3, 1:5, and 1:7) [43]. Then, probabilistic quotient normalization was used to remove the effects of dilution on the samples’ concentrations. The data tables thus generated underwent data analysis. More details are provided in Supplementary Materials Section S1.

The targeted methods were developed for the metabolites revealed by metabolic profilingand implemented using UPLC coupled to a triple-quadrupole mass spectrometer. Data acquisition and pre-processing were performed as reported in the Supplementary Materials Section S2.

### 4.4. Statistical Data Analysis

The recruited neonates’ demographic and perinatal data and laboratory findings were investigated applying t-test for normally distributed data, Mann–Whitney test for non-normally distributed data, and Fisher’s exact test for categorical data considering statistically significant tests with *p*-values less than 0.05. Normality was assessed using the Shapiro–Wilk test assuming normally distributed data for *p*-value > 0.10. Numerical data normally distributed have been reported as mean (standard deviation), numerical data non-normally distributed as median [interquartile range], and categorical data as the number of cases (percentage) with respect to the reference group.

Data emerging from untargeted and targeted metabolomics were investigated by both univariate and multivariate data analysis techniques. Untargeted data were mean-centered, whereas targeted data were auto-scaled prior to performing data analysis.

Univariate data analysis was based on the t-test in the case of normallydistributed data (*p*-value > 0.10 for the Shapiro–Wilk test) or the Mann–Whitney test in the presence of not normally distributed data (*p*-value < 0.10 for the Shapiro–Wilk test), controlling the false discovery rate at the level by the Benjamini–Hochberg procedure.

Multivariate data analysis was performed using projection methods. Specifically, Principal component analysis (PCA) was applied for outlier detection, while differences in the metabolic profiles of urine or plasma samples were investigated using projection to latent structures discriminant analysis (PLS–DA) with stability selection [29,44]. Class membership was assessed by applying linear discriminant analysis to the scores of the PLS–DA model built autoscaling the dummy variables specifying the class of the sample. Because structured noise was not detected in the models, variable influence on projection score (VIP) was used for ranking the predictors within the stability selection procedure. Overall, 500 subsets were extracted by bootstrap and relevant features were identified assuming a significance level equal to 0.05. Five-fold cross-validation was applied in model optimization. Matthew correlation coefficient in calculation (MCC) and MCC calculated by five-fold cross-validation (MCC_5-fold_) were used to measure the goodness-of-fit and to estimate the power in the prediction of the models. Moreover, a permutation test on the class response (1000 random permutations) was performed to highlight over-fitting and to estimate the *p*-values of MCC and MCC_5-fold_.

The relevant variables selected by multivariate data analysis were merged with those obtained from univariate data analysis and were annotated by searching our in-house database, the Human Metabolome Database, and the METLIN metabolite database. More details about variable annotation are provided in Supplementary Materials Section S1.3.

Over-representation pathway analysis was performed considering 80 pathways for *Homo sapiens*.

Data preprocessing and analyses were conducted using in-house R-functions implemented on the R 3.6.0 platform (R Foundation for Statistical Computing, Vienna, Austria). MetaboAnalyst 3.0 was adopted for the over-representation pathway analysis.

### 4.5. Ethical Approval

The study was approved by the Ethics Committee of Padua Hospital (protocol 3636/AO/15), and written informed consent was obtained from all parents/guardians before enrolling their child.

## 5. Conclusions

In conclusion, urine and plasma samples obtained from neonates with EOS at birth showed a distinctive metabolic profile that enabled them to be clearly distinguished from those without sepsis using UPLC–MS-based analysis. The tryptophan and glutathione metabolic pathways were severely disrupted in the septic neonates. The results of this proof-of-concept study support the potential of metabolomics for elucidating the biological pathways and pathophysiological mechanisms of sepsis. Upon validation, these findings could lay the foundations for the discovery of new early biomarkers and therapeutic targets of neonatal sepsis.

## Figures and Tables

**Table 1:** The recruited neonates’ demographic and perinatal characteristics, and laboratory findings at birth; numerical data normally distributed are reported as mean (standard deviation), whereas non-normally distributed data as median[interquartile range], and categorical data as the number of cases (percentage) with respect to the reference group.

Descriptive Variable	EOS Group (*n* = 15)	Controls (*n* = 15)	*p*-Value
Gestational age [days]	207 (17)	213 (16)	0.34
Birth weight [g]	1269 (358)	1300 (354)	0.82
Male sex (%)	7 (47)	5 (33)	0.71
Apgar score 1 [min]	7.0 [1.5]	7 [1]	0.32
Apgar score 5 [min]	8.0 [1.5]	8 [1]	0.11
Cesarean section	14 (93)	15 (100)	>0.99
Prenatal steroids	13 (87)	13 (87)	>0.99
Small for gestational age	2 (13)	6 (40)	0.21
Positive maternal vaginal swab	3 (20)	0 (0)	0.22
Premature rupture of membranes >18 h	4 (27)	2 (13)	0.65
Inotropes	0 (0)	1 (7)	>0.99
C-reactive protein <2.9 mg/L–at birth	12 (80)	15 (100)	0.22
White blood count-day 0 [K/μL]	4.9 [2.8]	8.2 [6.2]	0.07
Platelet count-day 0 [K/μL]	200 [83]	245 [70]	0.43

**Table 2:** Annotated variables. Type: CTRL > SEPSIS indicates that the mean was higher in the control group than in the earlyonset sepsis (EOS) group; SEPSIS > CTRL indicates that the mean was higher in the EOS group than in the control group. HMDB ID: identifier used in the Human Metabolome Database. Level: annotation level according to reference 30. AUC (CI 95%): confidence interval at the level of 95% of the area under the receiver operating characteristic (ROC) curve.

m/z	Type	HMDB ID	Level	Annotation	AUC (CI 95%)
175.0244	CTRL > SEPSIS	HMDB00044	2	Ascorbic acid	0.66–1.00
328.0445	CTRL > SEPSIS	HMDB00058	2	Cyclic AMP	0.53–1.00
132.0772	CTRL > SEPSIS	HMDB00064	2	Creatine	0.62–1.00
130.0868	CTRL > SEPSIS	HMDB00070	2	Pipecolic acid	0.58–1.00
152.0575	CTRL > SEPSIS	HMDB00132	1	Guanine	0.72–1.00
464.3013	CTRL > SEPSIS	HMDB00138	1	Glycocholic acid	0.66–1.00
137.0464	CTRL > SEPSIS	HMDB00157	2	Hypoxanthine	0.75–1.00
180.0655	CTRL > SEPSIS	HMDB00158	1	L-Tyrosine	0.60–1.00
164.0708	CTRL > SEPSIS	HMDB00159	1	L-Phenylalanine	0.76–1.00
90.0555	CTRL > SEPSIS	HMDB00161	2	L-Alanine	0.64–1.00
116.0711	CTRL > SEPSIS	HMDB00162	2	L-Proline	0.65–1.00
154.0618	CTRL > SEPSIS	HMDB00177	1	L-Histidine	0.60–1.00
147.1132	CTRL > SEPSIS	HMDB00182	1	L-Lysine	0.54–1.00
239.016	CTRL > SEPSIS	HMDB00192	1	L-Cystine	0.58–1.00
165.0552	CTRL > SEPSIS	HMDB00205	2	Phenylpyruvic acid	0.56–1.00
187.1084	CTRL > SEPSIS	HMDB00206	1	N6-Acetyl-L-lysine	0.75–1.00
335.068	CTRL > SEPSIS	HMDB00229	2	Nicotinamide ribotide	0.76–1.00
126.0224	CTRL > SEPSIS	HMDB00251	1	Taurine	0.70–1.00
283.0677	CTRL > SEPSIS	HMDB00299	2	Xanthosine	0.70–1.00
160.0607	CTRL > SEPSIS	HMDB00510	2	Aminoadipic acid	0.57–1.00
290.1607	CTRL > SEPSIS	HMDB00552	2	3-Methylglutarylcarnitine	0.38–0.95
120.0123	CTRL > SEPSIS	HMDB00574	1	L-Cysteine	0.61–1.00
135.0309	CTRL > SEPSIS	HMDB00613	2	Erythronic acid	0.80–1.00
269.0601	CTRL > SEPSIS	HMDB00676	2	L-Homocystine	0.35–0.94
609.2639	SEPSIS > CTRL	HMDB00683	2	Harderoporphyrin	0.62–1.00
209.0928	CTRL > SEPSIS	HMDB00684	1	L-Kynurenine	0.61–1.00
130.0867	CTRL > SEPSIS	HMDB00687	1	L-Leucine	0.65–1.00
246.1706	SEPSIS > CTRL	HMDB00688	2	Isovalerylcarnitine	0.62–1.00
130.0506	CTRL > SEPSIS	HMDB00725	1	Hydroxyproline	0.65–1.00
188.0712	CTRL > SEPSIS	HMDB00734	2	Indoleacrylic acid	0.64–1.00
192.0662	CTRL > SEPSIS	HMDB00763	2	5-Hydroxyindoleacetic acid	0.59–1.00
153.0413	CTRL > SEPSIS	HMDB00786	2	Oxypurinol	0.83–1.00
382.0995	CTRL > SEPSIS	HMDB00912	2	Succinyladenosine	0.61–1.00
158.0815	CTRL > SEPSIS	HMDB00927	2	Valerylglycine	0.66–1.00
203.0818	CTRL > SEPSIS	HMDB00929	1	L-Tryptophan	0.70–1.00
385.1296	CTRL > SEPSIS	HMDB00939	2	S-Adenosylhomocysteine	0.45–0.95
305.0977	SEPSIS > CTRL	HMDB01067	2	N-Acetylaspartylglutamic acid	0.50–0.97
153.0415	CTRL > SEPSIS	HMDB01182	2	6,8-Dihydroxypurine	0.85–1.00
219.1111	SEPSIS > CTRL	HMDB01238	2	N-Acetylserotonin	0.58–1.00
136.076	CTRL > SEPSIS	HMDB01250	2	N-Acetylarylamine	0.62–1.00
298.1126	SEPSIS > CTRL	HMDB01563	2	1-Methylguanosine	0.67–1.00
150.0556	CTRL > SEPSIS	HMDB01859	2	Acetaminophen	0.70–1.00
123.0446	CTRL > SEPSIS	HMDB01870	2	Benzoic acid	0.60–1.00
346.1228	SEPSIS > CTRL	HMDB01913	2	Omeprazole	0.47–0.98
86.0605	CTRL > SEPSIS	HMDB02039	2	2-Pyrrolidinone	0.65–1.00
329.175	CTRL > SEPSIS	HMDB02121	2	Carnosol	0.65–1.00
147.0444	CTRL > SEPSIS	HMDB02359	2	Phenylpropiolic acid	0.50–1.00
175.0606	SEPSIS > CTRL	HMDB03070	2	Shikimic acid	0.67–1.00
337.1276	SEPSIS > CTRL	HMDB03409	2	Berberine	0.54–1.00
179.0559	SEPSIS > CTRL	HMDB03466	2	L-Gulonolactone	0.49–1.00
301.1803	CTRL > SEPSIS	HMDB03955	2	19-Hydroxyandrost-4-ene-3,17-dione	0.65–1.00
232.028	CTRL > SEPSIS	HMDB04148	2	Dopamine 4-sulfate	0.63–1.00
257.0772	CTRL > SEPSIS	HMDB04813	2	3-Methyluridine	0.52–1.00
296.1395	SEPSIS > CTRL	HMDB05037	2	Sumatriptan	0.50–0.99
671.5588	CTRL > SEPSIS	HMDB05233	2	DG(20:1(11Z)/20:4(5Z,8Z,11Z,14Z)/0:0)[iso2]	0.60–1.00
290.1356	CTRL > SEPSIS	HMDB05765	2	Ophthalmic acid	0.41–0.96
296.0998	CTRL > SEPSIS	HMDB05862	2	2-Methylguanosine	0.53–1.00
73.0301	CTRL > SEPSIS	HMDB06112	2	Malondialdehyde	0.59–1.00
275.1128	SEPSIS > CTRL		3		0.80–1.00
819.2381	SEPSIS > CTRL		3		0.52–1.00

**Table 3:** Relevant metabolites for blood samples. SEPSIS > CTRL indicates that the mean in the EOS group is higher than in the control group. HMDB ID: identifier used in the Human Metabolome Database. AUC (CI 95%): confidence interval at the level of 95% of the area under the receiver operating characteristic (ROC) curve.

Name	HMDB ID	Type	AUC (CI 95%)
glycine	HMDB0000123	SEPSIS > CONTROL	0.59–0.96
tyrosine	HMDB0000158	SEPSIS > CONTROL	0.48–0.89
phenylalanine	HMDB0000159	SEPSIS > CONTROL	0.56–1.00
alanine	HMDB0000161	SEPSIS > CONTROL	0.50–0.92
proline	HMDB0000162	SEPSIS > CONTROL	0.52–0.95
asparagine	HMDB0000168	SEPSIS > CONTROL	0.49–0.88
lysine	HMDB0000182	SEPSIS > CONTROL	0.48–0.89
serine	HMDB0000187	SEPSIS > CONTROL	0.59–0.99
cystine	HMDB0000192	SEPSIS > CONTROL	0.52–0.92
ornithine	HMDB0000214	SEPSIS > CONTROL	0.59–0.97
serotonin	HMDB0000259	SEPSIS > CONTROL	0.49–0.88
sarcosine	HMDB0000271	SEPSIS > CONTROL	0.66–1.00
tryptamine	HMDB0000303	SEPSIS > CONTROL	0.51–0.91
tyramine	HMDB0000306	SEPSIS > CONTROL	0.51–0.91
aminoadipic acid	HMDB0000510	SEPSIS > CONTROL	0.52–0.93
kynurenine	HMDB0000684	SEPSIS > CONTROL	0.49–0.88
methionine	HMDB0000696	SEPSIS > CONTROL	0.49–0.922
kynurenic acid	HMDB0000715	SEPSIS > CONTROL	0.51–0.92
5-HIAA	HMDB0000763	SEPSIS > CONTROL	0.48–0.90
xanthurenic acid	HMDB0000881	SEPSIS > CONTROL	0.49–0.88
valine	HMDB0000883	SEPSIS > CONTROL	0.49–0.92
citrulline	HMDB0000904	SEPSIS > CONTROL	0.52–0.92
spermidine	HMDB0001257	SEPSIS > CONTROL	0.48–0.89
N1-AcetylSPD	HMDB0001276	SEPSIS > CONTROL	0.49–0.89
ADMA	HMDB0001539	SEPSIS > CONTROL	0.49–0.92
cadaverine	HMDB0002322	SEPSIS > CONTROL	0.64–0.98

## Data Availability

The data of the study are almost all presented in the study, but if someone will need some more information we will be pleased to provide them (at the moment these data are not in an on line publicly archived dataset).

## Supplementary Materials

The following are available online at https://www.mdpi.com/2218-1989/11/2/115/s1, Figure S1: NEG data set: score scatter plot (panel A) and T2/Q plot (panel B) obtained for the con-trols and score scatter plot (panel C) and T2/Q plot (panel D) obtained for the group of neonates developing sepsis, Figure S2: POS data set: score scatter plot (panel A) and T2/Q plot (panel B) obtained for the controls and score scatter plot (panel C) and T2/Q plot (panel D) obtained for the group of neonates developing sepsis, Table S1: The name of the 64 metabolites quantified by tar-geted methods, their chemical group, the LOQ concentration, the value of R2 for calibration curves, and the commercial name and the companies where we purchased the standards are reported, Table S2: The labeled standards used for calibration curves and the name of the chemical companies where we purchased the internal standards are reported, Section S1: Untargeted Metabolomic Profiling, Section S2: Targeted metabolomic analysis.
