# Supplementary material for: Untargeted and Targeted Metabolomic Profiling of Preterm Newborns with EarlyOnset Sepsis: A Case-Control Study
Source: Metabolites. 2021 Feb 18;11(2):115. doi: 10.3390/metabo11020115 (PMC7922887; doi:10.3390/metabo11020115)
Supplement: Supplementary file 1 [file metabolites-11-00115-s001.pdf]

SUPPLEMENTARY MATERIALS

S1 Untargeted metabolomic profilin

S1.1 LC-MS analysis

Untargeted metabolic profiling was performed in positive and negative ionization mode on an Acquity Ultra Performance Liquid Chromatography (UPLC) system (Waters, U.K.) coupled to a Quadrupole Time-of-Flight (QToF) Synapt G2 HDMS mass spectrometer (Waters MS Technologies, Ltd., Manchester, U.K.).

For LC-MS analysis a Waters Acquity UPLC HSS T3 column 2.1 mm wide and 100 mm long packed with 1.8 µm beads was used and its temperature was kept at 50 °C. The mobile phase flow rate was set at 0.5 ml/min. The gradient mobile phase consisted of water with 0.1% FA (A) and methanol with acetonitrile in a 90:10 ratio with 0.1% FA (B). Each sample run lasted 12 min and consisted of an isocratic phase of 5% B for 1 min, a linear increase to 30% B in 2.5 min, a linear increase to 95% B in 3 min, an isocratic phase of 95% B for 1.5 min, a washout phase of 5% B for 3 min. For each run, 5 µl of sample were injected.

Mass acquisition was performed with the Quadrupole-Time-of-Flight (Q-TOF) Mass Spectrometer (Synapt G2, Waters Co.) operating at both positive-ion (ESI+) and negative-ion (ESI-)electro-spray ionization mode. The mass range scan was of 20 to 1200 amu, both in MS scan mode and in MSe mode to obtain the fragmentation spectrum of the variables that fall within the parameters set in the scan method in MSe. The capillary voltage was set at 3.5 KV in positive mode and 2.8 KV in negative mode; the sampling cone voltage was set at 30 V in both modes. The desolvation gas flow was set at 600 L/h with temperature kept at 350 °C. The cone gas flow was set at 20 L/h with temperature kept at 110 °C. To correct for changes in environmental or experimental condition over the course of the analysis, Leucine-Enkephalin ($[M+H]^+$ = 556,2771 m/z and $[M-H]^-$ = 554,2615 m/z), at a concentration of 2 µg/ml in a solution of acetonitrile and water with 0.1% FA in a 50:50 ratio, was injected periodically (every 10 s) as internal reference (i.e. lock mass).

Quality control (QC) samples and standard solution samples were used to test for reproducibility and accuracy during the analysis and were injected at regular intervals throughout the sequence, together with blank samples. To further reduce analytical variability, in accordance with an in-house protocol, samples distribution in the plate and the sequence of sample injection in the UPLC-MS were randomized, and 5/6 of the fluid resulting from the addition of eluents to the sample was excluded from the ionization process (splitting). Splitting samples prevent the risk of smudge the internal surfaces of the spectrometer itself, thus reducing its sensitivity.

S1.2 Data processing and pre-treatment

UPLC-MS data were processed by the ProgenesisQI software (Waters Corporation, Milford, U.S.A.) and two data sets were generated, one for the positive-ionization mode (POS data set) and the other for the negative-ionization mode (NEG data set). The parameters used for data extraction were optimized through the preliminary analysis processing of the QC samples. We used a filter strength of 0.25 for import raw data and a QC in the middle of the sequence as a reference for the automatic alignment of all runs in the sequence. For the peak picking the sensitivity of the automatic algorithm was set at 3, where retention time

limits were between 0.4 and 8 min. The so-called Rt_mass variables (where Rt is the retention time and mass is the mass to charge ratio m/z of the chemical compound) were generated.

Variables with more than 20% of missing data were eliminated to avoid spurious statistical models generated by unrealistic combinations of the measured variables. For each variable passing such a filter, missing data were imputed with a random number between zero and the minimum value measured for the variable. Variables with a coefficient of variation greater than 20% for QC samples have been excluded. Variables detected in the blank samples have been subtracted to the samples. The ion intensities for each peak detected were normalized, based on the calibration models obtained for the QCs with different dilution factors (1:3, 1:5, 1:7). Then probabilistic quotient normalization was applied to take into account dilution effects.

S1.3 Variables annotation

The relevant variables selected by multivariate data analysis were merged with those obtained from univariate data analysis and were annotated by searching our in-house database of commercial standards, the METLIN metabolite database, and the Human Metabolome Database to obtain a unique identification code (HMDB ID)

Annotation for each putative marker was assigned with a different level of confidence (as described in Viant et al. Curr Opin Chem Biol. **2017**;36:64-69), based on accurate mass, retention time, and the fragmentation patterns, where available. To improve confidence in the compound annotation, for the compounds not present in our database, theoretical fragmentation of the candidate list of compounds was performed in Progenesis, and the resulting *in silico* fragmentation matched against the observed fragments for a compound.

Level 1 was assigned for the compounds with a difference of 10 ppm for m/z, 0.2 min for rt, and, where available, with collision cross-section ≥2%, with respect to the standards of our in-house database, that were performed under identical analytical conditions of the current analysis.

Instead, level 2 and 3 was for metabolites with m/z ≤10ppm respect to the online databases, and the fragmentation score ≥ 30 or < 30, respectively.

S1.4 Outlier detection

PCA has been applied to detect the presence of outliers in the untargeted metabolomic data obtained from urine samples. Specifically, T2 test and Q test have been applied with a significance level $\alpha=0.05$. Data have been mean-centered and 2 principal components have been considered. In figures S1 and S2 the score scatter plots and the T2/Q plots obtained respectively for the NEG and the POS data sets are reported.

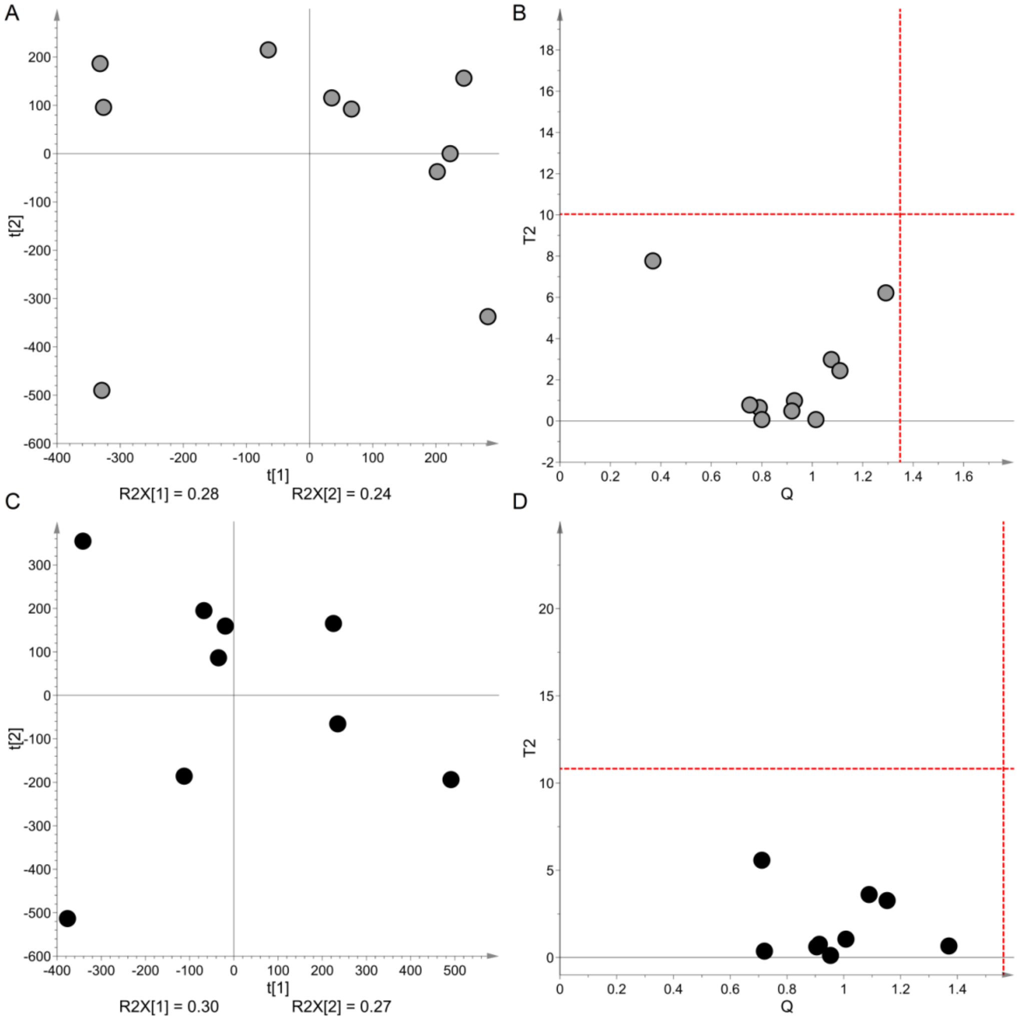

Figure S1. NEG data set: score scatter plot (panel A) and T2/Q plot (panel B) obtained for the controls and score scatter plot (panel C) and T2/Q plot (panel D) obtained for the group of neonates developing sepsis; red dashed lines indicate the threshold at the significance level α=0.05 used for outlier detection.

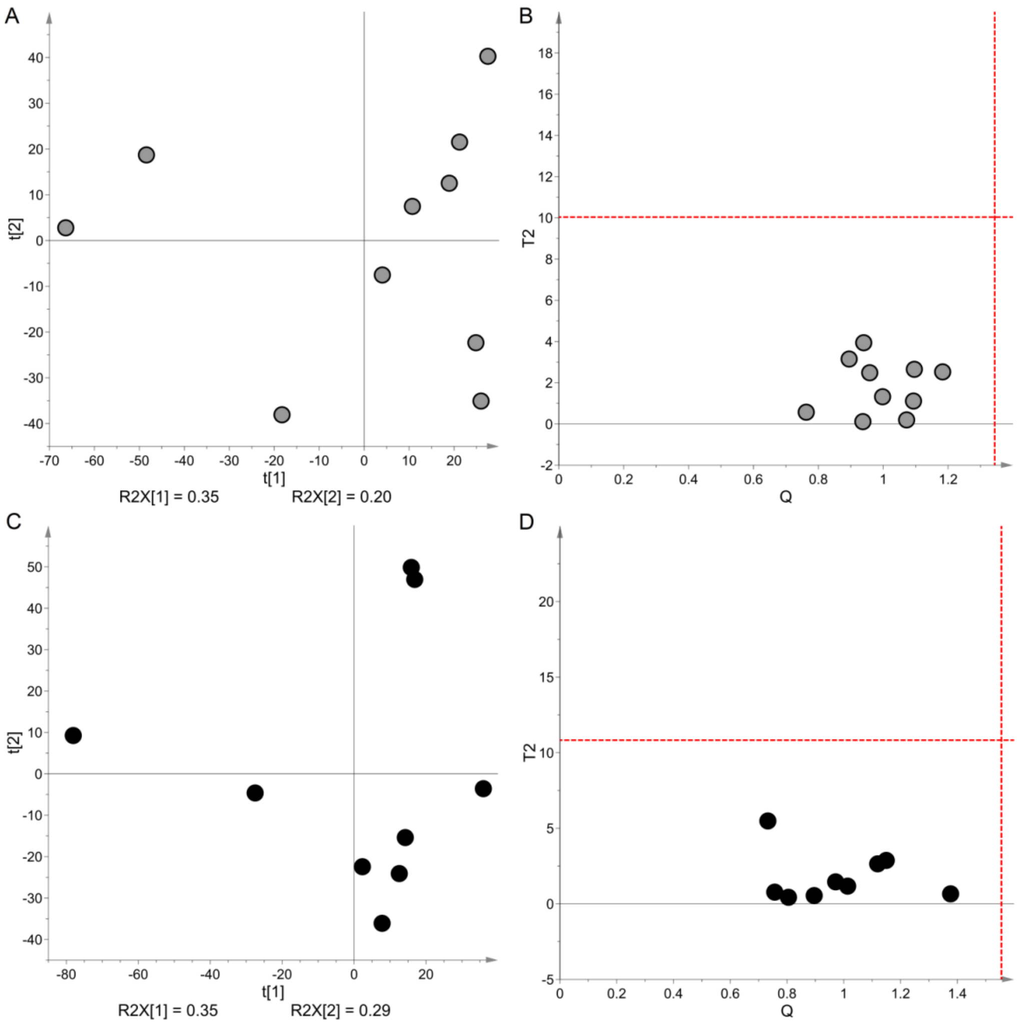

Figure S2. POS data set: score scatter plot (panel A) and T2/Q plot (panel B) obtained for the controls and score scatter plot (panel C) and T2/Q plot (panel D) obtained for the group of neonates developing sepsis; red dashed lines indicate the threshold at the significance level α=0.05 used for outlier detection.

S2 Targeted metabolomic analysis

S2.1 Chemicals and reagents

The chemical standards and labeled standards were purchased from: Sigma-Aldrich Corporation (Milan, Italy); Fluka (Milan, Italy); CDN Isotopes (Pointe-Claire, Quebec, Canada).; Chromsystems Instruments &Chemicals (Gräfelfing, Germany), Toronto Research Chemicals (Toronto, Ontario, Canada); Santa Cruz Biotechnology, Inc. (Dallas, Texas, USA); Coompo Research Chemicals (Wuhan, Hubei, PRC). The commercial names and the specific chemical company for each analyte were indicated in Table S1 and Table S2. The purity of all analytes and labeled internal standards was ≥98%.

Water was purified with a Milli-Q Elix purification system (Millipore, Bedford, MA, USA). High-purity MS-grade solvents (formic acid, methanol, and acetonitrile) were obtained from Fluka (Milan, Italy) and used without further purification.

S2.2 Preparation of standard solutions and calibration curves

Individual stock solutions in water or methanol with different percentage of formic acid depending on the different solubility of the compounds were used. A series of solution mixtures of desired concentrations were prepared by suitable dilutions of the stock solutions in 0.1% formic acid in water. All the stocks were stored at -20 °C.

Stock solutions of labeled metabolites were prepared as the unlabeled and diluted as required, with water 0.1% FA, to obtain a concentration of 0.05-0.1 μM for neurotransmitters, and polyamine, and 1-10 μM for amino acids and kynurenine metabolites, and used as internal standard (IS).

Calibration curves of the analytes were prepared by spiking pooled plasma, obtained from volunteers, with the diluted mixed standard solutions and IS, to the concentration ranging from 0.3 to 100 nmol/L for neurotransmitters, from 30 to 3000 nmol/L for polyamines, and from 0.05 to 250 μmol/L for amino acids and kynurenine metabolites.

S2.3 Quality controls (QC)

Two different concentrations of QC's plasma were used for precision and accuracy. Where available we used QC from chemical companies, with 2 different level concentration (Amino Acid Quality Control set, low and High, Kairos™, Waters Corporation, Milford, MS, USA), otherwise we prepared QC by spiking pooled plasma with 2 different concentration of the analytes.

The QC's plasma were extracted 2 times and analyzed 5 times within the same chromatographic run (n=10, intraday repeatability) and for 3 distinct days (n=30, between days reproducibility) to precision and reproducibility of the analytical method, expressed as coefficient of variation (CV%).

Difference between measured and expected values of QC's plasma samples (Bias%) was used to estimate the accuracy of the analysis

The analytes with CV% and Bias% ≤ 20% were considered for targeted analysis.

Plasma calibrations curve at 5 concentrations were built for assessing linearity, expressed as R^2.

Sensitivity, expressed as limit of quantification (LOQ, S/N≥10), was extrapolated by lowest point of calibration solution. The R^2 and LOQ for each compound were reported in Table S1

S2.4 Sample preparation

S2.4.1 Sample preparation for the analysis of amino acids, polyamines and metabolites of the kynurenine pathway

10 µL of plasma were mixed with 10 µL polyamine internal standard (IS), 10 µL kynurenine internal standard, and 100 µL amino acids internal standard mix in methanol 0.1% v/v formic acid.

The sample+IS mixtures were deproteinized and vortexed, then stored at -20 °C for 20 min and centrifuged at 13000 g for 7 min. For the analysis of the metabolites of the kynurenine pathway, 50µL of supernatant were transferred to a vial and injected to LC-MS.

For amino acid and polyamine analysis, 10 µL of the supernatant were mixed in a well plate with 70 µL of borate buffer and 20 µL of AccQ-Tag reagent, (AccQ-Tag Ultra Derivatization Kit, Waters Corporation)

then heated for 10 min at 55 °C for derivatization. The plate was placed under a stream of N_2 for 10 min to evaporate the acetonitrile of the reagent, then 20 µL of buffer or H_2O were added.

10 µL of the samples were diluted with 190 µL H_2O in another plate for high-concentration amino acid quantification.

S2.4.2 Sample preparation for the analysis of neurotransmitters associated with tyrosine and tryptophan metabolism

10 µL of plasma were mixed with 5 µL of IS and 100 µL of cold acetonitrile. The sample+IS mixtures were vortexed and stored at -20 °C for 20 min, then centrifuged at 13000 g for 7 min. 10 µL of the supernatant were mixed in a well plate with 70 µL of borate buffer and 20 µL of AccQ-Tag reagent, and heated for 10 min at 55 °C for derivatization. The plate was then placed under a stream of N_2 for 10 min to evaporate the acetonitrile of the reagent, then 20 µL of buffer or H_2O were added.

S2.5 UPLC-MS analysis

The analysis was conducted using a Xevo TQ-S triple-quadrupole mass spectrometer coupled to an Acquity UPLC (Waters Milford, MA, USA), interfaced with a source of Electrospray Ionization (ESI). The ESI was operated in the positive ion mode with multiple reaction monitoring (MRM). Chromatographic separation was done on a Waters Acquity UPLC HSS T3 2.1 x 100 mm 1.8 µm column (Waters Milford, MA, USA). Specific mobile phases and injection volumes were used for the different classes of metabolites as summarized below:

Amino acids

Mobile phases consisted of waters 0.1% formic acid for phase A and acetonitrile 0.1% formic acid for phase B. Injection volume 2 µL.

Time(min)	Flow Rate	%A	%B
0	0.6	96	4
0.5	0.6	96	4
2.5	0.6	90	10
5	0.6	72	28
6	0.6	5	95
7	0.6	5	95
7.1	0.6	96	4

Polyamines

Mobile phases consisted of waters 0.1% formic acid for phase A and acetonitrile:methanol 90:10 0.1% formic acid for phase B. Injection volume 20 µL.

Time(min)	Flow Rate	%A	%B
0	0.6	96	4
1	0.6	96	4
2.5	0.6	90	10
5	0.6	85	15
5.5	0.6	78	22
6	0.6	5	95
7	0.6	5	95
7.5	0.6	3	97
7.6	0.6	96	4
8.5	0.6	96	4

Metabolites of the kynurenine pathway

Mobile phases consisted of waters 0.1% formic acid for phase A and acetonitrile:methanol 90:10 0.1% formic acid for phase B. Injection volume 5 µL.

Time(min)	Flow Rate	%A	%B
0	0.3	98	2
2.6	0.3	65	35
3.5	0.3	40	60
4	0.3	10	90
4.5	0.6	10	90
4.51	0.6	10	90
5.5	0.6	10	90
6	0.6	98	2
6.9	0.6	98	2
7	0.3	98	2

Neurotransmitters associated to tyrosine and tryptophan metabolism

Mobile phases consisted of waters 0.1% formic acid for phase A and acetonitrile:methanol 90:10 0.1% formic acid for phase B. Injection volume 20 µL.

Time(min)	Flow Rate	%A	%B
0	0.6	99	1
0.5	0.6	99	1
1	0.6	96	4
3	0.6	90	10
5.5	0.6	72	28
6.5	0.6	5	95
7.5	0.6	5	95
8.01	0.6	99	1
8.5	0.6	99	1

Instrument control, data acquisition and analysis were managed with MassLynx software (version 4.1, Waters). Quantification was done using the TargetLynx function of the same software.

Table S1. The name of the 64 metabolites quantified by targeted methods, their chemical group, the LOQ concentration, the value of R^2 for calibration curves, and the commercial name and the companies where we purchased the standards are reported.

Metabolite	HMDB_ID	Commercial name	Company	group/pathway	LOQ	R2
2-aminobutyric acid	HMDB0000452	L-a-Amino-n-butyric Acid	Sigma-Aldrich	aminoacids	3.08 µmol/L	0.9841
3-aminobutyric acid	HMDB0031654	3-aminobutyric acid	Sigma-Aldrich	aminoacids	2.46 µmol/L	0.9819
3-aminoisobutyric acid	HMDB0003911	D,L-ß-Aminoisobutyric Acid	Sigma-Aldrich	aminoacids	3.08 µmol/L	1
3-methylhistidine	HMDB0000479	3-Methyl-L-histidine	Sigma-Aldrich	aminoacids	3.08 µmol/L	0.9999
ADMA	HMDB0001539	dimethylarginine-dihydrochloride	Sigma-Aldrich	aminoacids	0.04 µmol/L	0.9985
alanine	HMDB0000161	L-Alanine	Sigma-Aldrich	aminoacids	3.08 µmol/L	0.9708
aminoadipic acid	HMDB0000510	L-a-Aminoadipic Acid	Sigma-Aldrich	aminoacids	3.08 µmol/L	0.9909
3-aminopropanoic acid		ß-Alanine	Sigma-Aldrich	aminoacids	3.08 µmol/L	0.9903
arginine	HMDB0000517	L-Arginine	Sigma-Aldrich	aminoacids	3.08 µmol/L	0.9952
asparagine	HMDB0000168	L-Asparagine	Sigma-Aldrich	aminoacids	3.08 µmol/L	0.9924
Aspartic	HMDB0000191	L-Aspartic Acid	Sigma-Aldrich	aminoacids	3.08 µmol/L	0.9993
carnosine	HMDB0000033	L-Carnosine	Sigma-Aldrich	aminoacids	3.08 µmol/L	0.9958
citrulline	HMDB0000904	L-Citrulline	Sigma-Aldrich	aminoacids	3.08 µmol/L	0.9996
cystathionine	HMDB0000099	Cystathionine*	Sigma-Aldrich	aminoacids	3.08 µmol/L	0.9987
Cystine	HMDB0000192	L-Cystine	Sigma-Aldrich	aminoacids	1.54 µmol/L	0.9976
dl-kinurenine	HMDB0000684	L-Kynurenine	Sigma-Aldrich	aminoacids/kynurenine pathways	0.12 µmol/L	0.9873
Ethanolamine	HMDB0000149	Ethanolamine	Sigma-Aldrich	aminoacids	3.08 µmol/L	0.9958
GABA	HMDB0000112	g-Amino-n-butyric Acid	Sigma-Aldrich	aminoacids	3.08 µmol/L	0.9875

glutamic acid	HMDB0000148	L-Glutamic Acid	Sigma-Aldrich	aminoacids	3.08 µmol/L	0.9974
glycine	HMDB0000123	Glycine	Sigma-Aldrich	aminoacids	3.08 µmol/L	0.9918
HArg	HMDB0000670	L-homoarginine hydrochloride	Fluka	aminoacids	0.04 µmol/L	0.9985
histidine	HMDB0000177	L-Histidine	Sigma-Aldrich	aminoacids	3.08 µmol/L	0.9981
homoserine	HMDB0000719	L-homoserine	Fluka	aminoacids	2.46 µmol/L	0.9922
isoleucine	HMDB0000172	L-Isoleucine	Sigma-Aldrich	aminoacids	3.08 µmol/L	0.997
leucine	HMDB0000687	L-Leucine	Sigma-Aldrich	aminoacids	3.08 µmol/L	0.9995
lysine	HMDB0000182	L-Lysine	Sigma-Aldrich	aminoacids	3.08 µmol/L	0.9869
methionine	HMDB0000696	L-Methionine	Sigma-Aldrich	aminoacids	3.08 µmol/L	0.9988
NMMA		N-monomethyl-L-arginine	Sigma-Aldrich	aminoacids	0.04 µmol/L	0.9998
OH-lysine/allo-OH-lysine	HMDB0000450	d-DL-Hydroxylysine	Sigma-Aldrich	aminoacids	3.08 µmol/L	0.991
ornithine	HMDB0000214	L-Ornithine	Sigma-Aldrich	aminoacids	3.08 µmol/L	0.9821
phenylalanine	HMDB0000159	L-Phenylalanine	Sigma-Aldrich	aminoacids	3.08 µmol/L	0.998
proline	HMDB0000162	L-Proline	Sigma-Aldrich	aminoacids	3.08 µmol/L	0.9926
sarcosine	HMDB0000271	L-Sarcosine	Sigma-Aldrich	aminoacids	3.08 µmol/L	0.9987
SDMA	HMDB0003334	dimethyl-L-arginine-di(p-hydroxyazobenzene-p-sulfonate)	Sigma-Aldrich	aminoacids	0.04 µmol/L	0.9976
serine	HMDB0000187	L-Serine	Sigma-Aldrich	aminoacids	3.08 µmol/L	0.9906
taurine	HMDB0000251	Taurine	Sigma-Aldrich	aminoacids	3.08 µmol/L	0.9963
threonine	HMDB0000167	L-Threonine	Sigma-Aldrich	aminoacids	3.08 µmol/L	0.9988
tryptophane	HMDB0000929	L-Tryptophan	Sigma-Aldrich	aminoacids/kynurenine pathways	3.08 µmol/L	0.9943
tyrosine	HMDB0000158	L-Tyrosine	Sigma-Aldrich	aminoacids	3.08 µmol/L	0.9994
valine	HMDB0000883	L-Valine	Sigma-Aldrich	aminoacids	3.08 µmol/L	0.9955
3-HAA	HMDB0001476	3-Hydroxyanthranilic acid	Sigma-Aldrich	kynurenine pathways	0.01 µmol/L	0.995
3OH-KYN	HMDB0011631	3-Hydroxy-DL-kynurenine	Sigma-Aldrich	kynurenine pathways	0.01 µmol/L	0.9974
5-HIAA	HMDB0000763	5-Hydroxyindole-3-acetic acid	Sigma-Aldrich	kynurenine pathways	0.01 µmol/L	0.9888
5-OH-ind	HMDB0001855	5-Hydroxyindole	Sigma-Aldrich	kynurenine pathways	0.05 µmol/L	0.9997
IAA	HMDB0000197	3-Indoleacetic acid	Sigma-Aldrich	kynurenine pathways	0.05 µmol/L	0.9899
IPA	HMDB0002302	Indole-3-propionic acid	Sigma-Aldrich	kynurenine pathways	0.05 µmol/L	0.9918
KYNA	HMDB0000715	kynurenic acid	Sigma-Aldrich	kynurenine pathways	0.02 µmol/L	0.9706
XA	HMDB0000881	xanthurenic acid	Sigma-Aldrich	kynurenine pathways	0.004 µmol/L	0.9719
agmatine	HMDB0001432	Agmatine sulfate salt	Sigma-Aldrich	polyamine	0.12 µmol/L	0.9931
cadaverine	HMDB0002322	Cadaverine dihydrochloride	Sigma-Aldrich	polyamine	0.09 µmol/L	0.9986
N1-AcetylSPD	HMDB0001276	N1-acetylspermidine (hydrochloride)	Cayman Chemicals	polyamine	0.03 µmol/L	0.9958
putrescine	HMDB0001414	Putrescine dihydrochloride	Sigma-Aldrich	polyamine	0.03 µmol/L	0.9922
spermidine	HMDB0001257	Spermidine trihydrocholride	Sigma-Aldrich	polyamine	0.03 µmol/L	0.9948
Spermine	HMDB0001256	Spermine tetrehydrocholride	Sigma-Aldrich	polyamine	0.06 µmol/L	0.9993
Dopamine	HMDB0000073	3-Hydroxy-Tyramine HCL	Sigma-Aldrich	neurotrasmitters	1.56 nmol/L	0.9701
epinephrine	HMDB0000068	(-) Epinephrine	Sigma-Aldrich	neurotrasmitters	1.56 nmol/L	0.9879
Metanephrine	HMDB0004063	D,L-Metanephrine Hydrochloride	Sigma-Aldrich	neurotrasmitters	1.56 nmol/L	0.9982
norepinephrine	HMDB0000216	DL-Noroadrenaline	Fluka	neurotrasmitters	2.5 nmol/L	0.9976
Octopamine	HMDB0004825	(±)-Octopamine hydrochloride	Sigma-Aldrich	neurotrasmitters	1.25 nmol/L	0.9963
Serotonin	HMDB0000259	5-Hydroxytyramine hydrochloride	Sigma-Aldrich	neurotrasmitters	0.62 µmol/L	0.9963

Synephrine	HMDB0004826	(±)-Synephrine	Sigma-Aldrich	neurotrasmitters	0.31 nmol/L	0.9918
Tryptamine	HMDB0000303	Tryptamine	Sigma-Aldrich	neurotrasmitters	3.12 nmol/L	0.9984
Tyramine	HMDB0000306	4-Hydroxyphenethylamine	Sigma-Aldrich	neurotrasmitters	3.12 nmol/L	0.9807

Table S2. The labeled standards used for calibration curves and the name of the chemical companies where we purchased the internal standards are reported.

Labeled standards	Company
Histidine D3	Sigma-Aldrich
Glycine 13C215N	Chromsystem
Arginine D7	Chromsystem
Glutamine D5	Sigma-Aldrich
Serine D3	CDN isotope
Aspartic Acid D3	Chromsystem
Citrulline D2	Chromsystem
Glutamic acid D5	Chromsystem
Alanine D4	Chromsystem
GABA D6	CDN isotope
Ornithine d6	Chromsystem
Proline D7	Sigma-Aldrich
Lysine D4	Sigma-Aldrich
Tyrosine D4	Chromsystem
Methionine D3	Chromsystem
Valine D8	Chromsystem
Leucine D3	Chromsystem
Phenylalanine D5	Chromsystem
Tryptophane D5	Sigma-Aldrich
Creatinine D3	CDN isotopes
Creatine-D3	CDN isotopes
Taurine D4	Sigma-Aldrich
5HIAA-D5	Sigma-Aldrich
L-Dopa-D3	CDN isotope
Agmatine D8	Coompo
Putrescine D4	CDN isotopes
Cadaverine D4	CDN isotopes
Spermidine 13C4	Sigma-Aldrich
Spermine D8	Sigma-Aldrich
Kynurenine-D4	Toronto Chemicals
Histamine D4	CDN isotopes
2-phenyl-d5-ethylamine (β-PEA-D5)	CDN isotopes
Octopamine D4	CDN isotopes
Dopamine D4	CDN isotopes
Synephrine 13C215N	Santa Cruz Biotech
Norepinephrine D6	CDN isotopes
Serotonin D4	CDN isotopes
Epinephrine D6	CDN isotopes
Metanephrine D3	Sigma-Aldrich
Tryptamine D4	CDN isotopes
Serotonin D4	CDN isotopes
